# Temporal trends in the clinical presentation, management, and outcomes of children with congenital cytomegalovirus infection

**DOI:** 10.1007/s00431-026-07485-2

**Published:** 2026-10-14

**Authors:** Jitka Bolchová, Despoina Gkentzi, Roberto Pedrero Tomé, Garyfallia Syridou, Antoni Noguera-Julián, María Ríos-Barnés, Serena Salomé, Francesco Raimondi, Serena Villaverde, Fernando Baquero-Artigao, Paula Rodriguez-Molino, Danilo Buonsenso, Laura Martino, Beatriz Álvarez-Vallejo, Jesús Saavedra, Yves Fougere, Hermione Lyall, Ina Foulon, Horst Buxmann, Simona Zanette, Olga Tsiatsiou, Jessica Wan, Daniel Blázquez-Gamero, Vana Papaevangelou, cCMVnet Registry Study Group

**Affiliations:** 1 Clinic of Infectious Diseases and Pediatric Department, Bulovka University Hospital, Prague, Czech Republic; 2 Third Faculty of Medicine, Charles University, Prague, Czech Republic; 3 Second Faculty of Medicine, Charles University, Prague, Czech Republic; 4 Department of Paediatric Infectious Diseases, Medical School, University of Patras, Patras, Greece; 5 Malalties Infecciosesi Resposta Inflamatòria Sistèmicaen Pediatria, Servei de Malalties Infecciosesi Patologia Importada, Institut de Recerca Pediàtrica Sant Joan de Déu, Barcelona, Spain; 6 Division of Neonatology, Department of Translational Medical Sciences, University of Naples Federico II, Naples, Italy; 7 Hospital Universitario La Paz, Instituto Investigación Hospital La Paz (IDIPaz), Madrid, Spain; 8 Hospital Fondazione Policlinico Universitario A. Gemelli IRCCS, Roma, Italy; 9 Pediatric Infectious Diseases and Immunodeficiencies Unit, Children’s Hospital, Vall d’Hebron Barcelona Hospital Campus, Barcelona, Spain; 10 Hospital Universitario Gregorio Marañón, Madrid, Spain; 11 Unit of Pediatric Infectious Diseases and Vaccinology, Department of Woman Mother and Child, Lausanne University Hospital, Lausanne, Switzerland; 12 Imperial College Healthcare NHS Trust, London, UK; 13 Department of Otorhinolaryngology and Head & Neck Surgery, Vrije Universiteit Brussels, University Hospital UZ Brussel, Brussels Health Campus, De Poolster, Rehabilitation Centre, Brussels, Belgium; 14 Main-Kinzig Clinics Gelnhausen, Gelnhausen, Germany; 15 Hospital Fondazione IRCCS Policlinico S. Matteo, Pavia, Italy; 16 Hippokration General Hospital of Thessaloniki, Thessaloniki, Greece; 17 Manchester University NHS Foundation Trust, Manchester, UK; 18 Pediatric Infectious Diseases Unit, Hospital Universitario 12 de Octubre, Madrid, Spain; 19 CIBERINFEC: Centro de Investigación Biomédica en Red Enfermedades Infecciosas, Madrid, Spain; 20 Universidad Complutense de Madrid, Madrid, Spain; 21 Instituto de Investigación Hospital, 12 de Octubre (i+12), Madrid, Spain; 22 Third Department of Pediatrics, National and Kapodistrian University of Athens, ATTIKON General Hospital, Athens, Greece

**Keywords:** Cytomegalovirus, Congenital, Outcome, Europe

## Abstract

Congenital cytomegalovirus (cCMV) is the most common congenital infection and a leading cause of sensorineural hearing loss as well as neurodevelopmental impairment. In recent years, awareness, screening, and management strategies for cCMV have evolved. This study aimed to assess temporal changes in the clinical presentation, treatment, and outcomes of children diagnosed with cCMV infection. We conducted a retrospective cohort analysis using data from the CCMVnet registry (www.ccmvnet.org), an international multicenter database collecting anonymized data on children with confirmed cCMV infection. A total of 1653 patients diagnosed between 2011 and 2024 were included and divided into two cohorts: 2011–2016 (*n* = 566) and 2017–2024 (*n* = 1087). The year 2017 was selected as a pragmatic cutoff, corresponding to the year of publication of the European expert consensus statement on congenital CMV. Children were identified through antenatal, clinical, hearing-related, and newborn screening pathways. Symptomatic cCMV at birth was defined by the presence of at least one predefined clinical, laboratory, hearing, or neuroimaging finding based on previously published criteria. A comparative analysis between two distinct time periods was conducted, encompassing neonatal clinical findings, neuroimaging results, treatment strategies, and long-term outcomes. Newborns classified as symptomatic at birth decreased in the more recent cohort, from 49.2% in P1 to 44.2% in P2 (*p* = 0.07). The use of intravenous ganciclovir decreased from 7.3% to 2.8% (*p* < 0.001), while the use of oral valganciclovir alone increased from 43.8% to 78.7% (*p* < 0.001). Differences in long-term outcomes were observed between the two periods, with motor impairment at 12 months decreasing from 16.5% to 6.2% (*p* < 0.001) and any long-term sequelae at 36 months decreasing from 40.0% to 31.0% (*p* = 0.05).

*Conclusion*: This study highlights temporal differences in the clinical spectrum, management, and outcomes of children with congenital CMV infection in Europe. The higher proportion of asymptomatic and mildly symptomatic children in the latter period may reflect changes in maternal and neonatal screening practices. The observed temporal differences provide insight into the evolving clinical landscape of cCMV diagnosis and management, while their underlying causes cannot be determined from the present data.

**Table:** 

**What is Known:** • *Congenital cytomegalovirus (cCMV) infection is the leading infectious cause of sensorineural hearing loss and neurodevelopmental impairment in children.* • *Clinical management of cCMV has evolved substantially over the last decade, particularly following the publication of the European expert consensus statement in 2017 and alongside expanding screening strategies.*	
**What is New:** • *In our cohort, children diagnosed with cCMV after 2017 presented with milder clinical manifestations and experienced fewer long-term sequelae than those diagnosed before 2017.* • *Temporal changes in diagnostic pathways, treatment practices, and clinical outcomes were observed between the two study periods.*

**What is Known:**

• *Congenital cytomegalovirus (cCMV) infection is the leading infectious cause of sensorineural hearing loss and neurodevelopmental impairment in children.*

• *Clinical management of cCMV has evolved substantially over the last decade, particularly following the publication of the European expert consensus statement in 2017 and alongside expanding screening strategies.*

**What is New:**

• *In our cohort, children diagnosed with cCMV after 2017 presented with milder clinical manifestations and experienced fewer long-term sequelae than those diagnosed before 2017.*

• *Temporal changes in diagnostic pathways, treatment practices, and clinical outcomes were observed between the two study periods.*

## Introduction

Congenital cytomegalovirus (cCMV) infection is the most common congenital infection worldwide and the leading infectious cause of sensorineural hearing loss (SNHL) as well as neurodevelopmental delay [1, 2]. The Congenital Cytomegalovirus Network (cCMVNET) is a European initiative established in 2020 to enhance the understanding, diagnosis, and management of congenital cytomegalovirus (cCMV) infection. Central to this initiative is the cCMVnet registry, a web-based REDCap database that collects anonymized, prospective data on children diagnosed with cCMV across 12 European countries. The present study is based on a retrospective analysis of anonymized patient data collected within the cCMVnet registry, aiming to assess temporal changes in the presentation, management, and outcomes of children diagnosed with cCMV in Europe over two distinct periods: 2011–2016 and 2017–2024. The rationale behind this time frame is the publication of the first European Expert Consensus Statement on Diagnosis and Management of cCMV in 2017 [3].

## Methods

This was a retrospective observational study performed in a prospective cohort (cCMVnet registry). All 1653 patients with cCMV in the database born between 1 st January 2011 and 31 st December 2024 were included. Children with confirmed cCMV infection (positive polymerase chain reaction (PCR) in urine, blood, or cerebrospinal fluid within the first 21 days of life) followed at participating centers were offered inclusion in the registry. Anonymized clinical information was stored using the REDCap data collection system. REDCap is hosted on a secure online server located at the Instituto de Investigación Hospital 12 de Octubre. The cCMVnet registry has been approved by the reference Ethics Committee at Hospital Universitario 12 de Octubre (IRB number 20/495) and by the ethics committees of all participating centers. The study was conducted in accordance with the ethical standards of the institutional and/or national research committees and with the 1964 Declaration of Helsinki and its later amendments or comparable ethical standards. Written informed consent was obtained from the parents or legal guardians of all participants at the time of inclusion in the registry. The cCMVnet registry includes patients from 12 European countries: Spain, Italy, Greece, the UK, Switzerland, Belgium, Portugal, the Czech Republic, Poland, Bulgaria, Germany, and Slovenia. The majority of patients were enrolled from Spain, followed by Italy and Greece.

To assess temporal trends, the study period was divided into two cohorts: 2011–2016 (P1, *n* = 566) and 2017–2024 (P2, *n* = 1087). The year 2017 was selected as a pragmatic cutoff, approximately separating the study period into two cohorts of sufficient size for comparison and corresponding to the year of publication of the European expert consensus statement on congenital CMV [3].

All children were evaluated at birth by a pediatrician and/or neonatologist looking for symptoms and signs of cCMV infection. Symptomatic cCMV at birth was defined according to the predefined criteria used in the cCMVnet registry, based on previously published criteria (Dreher et al., J Pediatr 2014; Pignatelli et al., Clin Infect Dis 2010). Hearing screening was conducted for all newborns at participating centers using automated auditory brainstem response (A-ABR) or otoacoustic emissions. Cranial ultrasound and/or MRI were performed according to local clinical practice, and the performance and findings of these examinations were recorded in the registry. No standardized radiological imaging protocol or centralized radiological review was used across participating centers. Infants were followed at 6 and 12 months of age and then yearly up to 5 years of age. In follow-up visits, motor impairment (paresis, spasticity), epilepsy, and neurodevelopmental delays were evaluated.

### Statistical analysis

All statistical analyses were performed using SPSS version 22. Categorical variables are shown as frequencies and percentages. Continuous variables are reported as mean and standard deviation (SD) or median and interquartile range [IQR], depending on distributional assumptions. Normality was assessed by the Shapiro–Wilk test. Between-group comparisons were made using Pearson’s chi-square or Fisher’s exact test for categorical variables, and Student’s *t*-test or the Mann–Whitney *U* test for continuous variables, as appropriate. For all hypothesis tests, a two-tailed *p*-value less than 0.05 was considered statistically significant.

## Definitions

1. cCMV was defined as the presence of a positive CMV-PCR in urine, blood, or cerebrospinal fluid (CSF) or CMV isolation in urine cultures in the first 21 days of life [4].
2. Microcephaly: Head circumference Z-score <  − 2 according to the reference charts adjusted by sex and gestational age [5].
3. Symptomatic cCMV: Infants were categorized as symptomatic if they presented with at least one of the following predefined clinical, laboratory, hearing, or neuroimaging findings used in the cCMVnet registry: abnormal physical examination at birth (petechiae, splenomegaly, hepatomegaly, jaundice, hypotonia, lethargy, seizures, poor sucking reflexes, motor paresis, microcephaly (head circumference <  − 2 Z-score head circumference), SNHL, small for gestational age (birth weight <  − 2 Z-score for gestational age), chorioretinitis, thrombocytopenia (< 100.000), ALT >  = 80 UI/dL, abnormalities in imaging of the central nervous system (white matter abnormalities (WMA), ventricular adhesions, intracranial calcifications, ventriculomegaly, cerebellar hypoplasia, periventricular cysts, caudothalamic/subependymal cysts, hydrocephalus, lissencephaly, polymicrogyria, and other migration abnormalities). The criteria used in the registry were based on previously published criteria (Dreher et al., J Pediatr 2014; Pignatelli et al., Clin Infect Dis 2010). Children with isolated lenticulostriate vasculopathy (LSV) were not classified as symptomatic.
4. SNHL was defined as the presence of hearing thresholds of > 30 dBnHL in ABR in any ear or if the pure-tone average is above a threshold of 20 dBHL in any ear, in the absence of middle ear disease, as excluded by normal (high-frequency) tympanometry and microscopic ear examination. Quantification of the degree of SNHL was performed. According to the classification of Bureau International d’Audiophonologie (BIAP 1996), being mild hearing loss pure-tone average between 21 and 40, moderate 41–70, severe 71–90, profound > 90 dB [6].
5. Sequelae were defined as the presence of any of the following: SNHL, visual impairment, epilepsy, and motor impairment.

## Results

In total, 1653 infants were included; 566 in Period 1 (P1) (2011–2016) and 1087 in Period 2 (P2) (2017–2024). Table 1 presents neonatal and follow-up data on included children. The reasons for cCMV diagnosis differed between the two study periods (*p* < 0.001). In both periods, infants were identified through a range of antenatal, clinical, hearing-related, and newborn screening pathways. Maternal seroconversion was the most frequently reported reason for diagnosis, increasing from 25.4% in P1 to 35.0% in P2, while hearing loss as a reason for diagnosis decreased from 9.5% to 4.3%. The proportion of infants identified following failed newborn hearing screening increased from 2.7% to 5.0%, and those identified through universal newborn screening increased from 1.1% to 2.7% (Table 2). The proportion of newborns classified as symptomatic at birth decreased in the more recent cohort, from 49.2% in P1 to 44.2% in P2 (*p* = 0.07).

**Table 1:** Postnatal findings of included infants

Newborn	P1	P2	*p*-value
Symptomatic at birth (*N*/total; %)	267/543; 49.2	457/1033; 44.2	0.07
Abnormal physical examination at birth (*N*; %)	158/529; 29.9	203/1019; 19.9	< 0.001
The median head circumference at birth	33.0 cm [IQR 31.0–34.0]	33.5 cm [IQR 32.0–34.7]	< 0.001
Splenomegaly (*N*; %)	30/529; 5.7	32/1019; 3.1	0.023
Hepatomegaly (*N*; %)	31/529; 5.9	33/1019; 3.2	0.020
Petechiae (*N*; %)	57/529; 10.8	68/1019; 6.7	0.007
Abnormal laboratory findings	104/398; 26.1	155/815; 19	0.006
Unilateral hearing loss right ear (*N*; %) left ear (*N*; %)	120/479; 25 128/480; 26	168/886; 19 168/885; 19	0.015, 0.001
Bilateral hearing loss (*N*; %)	189/516; 36.6	252/910; 27.7	0.001
Chorioretinitis (*N*; %)	7/515; 1.4	9/945; 1	0.652
First cranial US			
Abnormal first cranial US (*N*; %)	197/484; 40.7	383/934; 41,0	0.957
Focal or multifocal white matter abnormalities (*N*; %)	34/484; 7.0	34/934; 3.6	0.007
Single intracranial calcifications (*N*; %)	38/484; 7.9	15/934; 1.6	< 0.001
Multiple intracranial calcifications (*N*; %)	12/484; 2.5	47/934; 5.0	0.032
Periventricular cysts (*N*; %)	53/481; 11.0	57/934; 6.1	0.002
Caudothalamic or subependymal cysts (*N*; %)	15/484; 3.1	126/934; 13.5	< 0.001
First cranial MRI			
Abnormal MRI findings (*N*; %)	172/269; 63.9	359/711; 50.5	< 0.001
Focal or multifocal white matter abnormalities (*N*; %)	107/269; 39.8	153/711; 21.5	< 0.001
Single intracranial calcifications (*N*; %)	28/269; 10.4	12/711; 1.7	< 0.001
Mild to moderate ventriculomegaly (*N*; %)	35/269; 13.0	55/711; 7.7	0.015
Periventricular cysts (*N*; %)	49/269; 18.2	67/711; 9.4	< 0.001
Migration abnormalities (*N*; %)	23/269; 8.6	11/711; 1.5	< 0.001
Infant treatment			
Antiviral treatment (*N*; %)	339/544; 62.3	472/987; 47.8	< 0.001
Intravenous ganciclovir (*N*/total treated; %)	24/331; 7.3	13/469; 2.8	< 0.001
Intravenous ganciclovir duration	18.0 days [IQR 11.0–31.0]	12.0 days [IQR 6.0–23.0]	< 0.001
Oral valganciclovir duration (*N*; %)	162.0 days [IQR 56.5–182.0]	181.0 days [IQR 150.0–184.0]	< 0.001
Follow-up 6 months			
Motor impairment (*N*; %)	60/426; 14.1	44/723; 6.1	< 0.001
Hearing loss (*N*; %)	122/416; 29.3	176/725; 24.3	0.072
Any long-term sequelae (*N*; %)	120/374; 32.1	170/667; 25.5	0.027
Follow-up 12 months			
Motor impairment (*N*; %)	67/407; 16.5	38/616; 6.2	< 0.001
Hearing loss (*N*; %)	132/401; 32.9	155/613; 25.3	0.01
Epilepsy under treatment (*N*; %)	16/392; 4.1	11/621; 1.8	0.043
Visual impairment (*N*; %)	15/381; 3.9	9/605; 1.5	0.027
Any long-term sequelae (*N*; %)	140/360; 38.9	152/565; 26.9	< 0.001
Follow-up 24 months			
Motor impairment (*N*; %)	62/385; 16.1%	30/392; 7.7%	< 0.001
Epilepsy (*N*; %)	21/380; 5.5%	9/395; 2.3%	0.031
Any long-term sequelae (*N*; %)	131/350; 37.4	107/365; 29.3	0.026
Follow-up 36 months			
Motor impairment (*N*; %)	51/314; 16.2	19/227; 8.4	0.01
Epilepsy (*N*; %)	22/317; 6.9	6/234; 2.6	0.034
Any long-term sequelae (*N*; %)	120/300; 40.0	63/203; 31.0	0.05

*P1*, 2011–2016; *P2*, 2017–2024; *IQR* Interquartile range

**Table 2:** Reason for cCMV diagnosis

	P1 ***N*** (%)	P2 ***N*** (%)	***p***-value
Maternal seroconversion during pregnancy	67 (25.4)	377 (35.0)	
Positive IgG and IgM with low avidity index (pregnancy)	16 (6.1)	57 (5.3)	
Antenatal fetal US abnormalities (pregnancy)	15 (5.7)	61 (5.7)	
Maternal symptomatic infection (pregnancy)	17 (6.4)	48 (4.5)	
Positive PCR in amniocentesis	11 (4.2)	39 (3.6)	
Failed newborn hearing screening test	7 (2.7)	54 (5.0)	
Clinical symptoms/signs consistent with cCMV at birth	35 (13.3)	165 (15.3)	
Hearing loss	25 (9.5)	46 (4.3)	
Neurologic abnormalities	7 (2.7)	30 (2.8)	
Prematurity	12 (4.5)	43 (4.0)	
Screening of baby born to mother with HIV	9 (3.4)	9 (0.8)	
Newborn universal screening program	3 (1.1)	29 (2.7)	
Other	40 (15.2)	120 (11.1)	
			*p* < 0.001

^*^Multiple reasons for cCMV diagnosis could be reported for individual patients

Among symptomatic newborns, the most commonly observed findings included petechiae, jaundice, hepatosplenomegaly, microcephaly, neurological abnormalities, and hearing loss. The median head circumference at birth increased significantly in the later cohort, from 33.0 cm in P1 to 33.5 cm in P2 (*p* < 0.001). Classical clinical signs such as splenomegaly, hepatomegaly, and petechiae were significantly less frequent in newborns diagnosed after 2016 (Table). A smaller proportion of newborns had an abnormal physical examination at birth or presented with abnormal blood tests in the later cohort, decreasing from 29.9% in P1 to 19.9% in P2 (*p* < 0.001) and 26.1% vs. 19.0%, (*p* = 0.006), respectively. Additionally, SNHL confirmed after newborn screening was significantly more common in P1: any form of hearing loss (mild/moderate/severe/profound) on right/left ear was detected in 25.1%/26.7% in P1 compared to 19%/19% in P2, *p* = 0.015, and bilateral hearing loss declined from 36.6% to 27.7%, *p* = 0.001.

## Neonatal cranial ultrasound

The proportion of newborns who underwent cranial ultrasound remained stable over time, at 85% prior to 2017 (484/566) and 86% (934/1087) thereafter. Neuroimaging findings from the initial cranial ultrasound (cUS) performed in the neonatal period revealed a significant reduction in abnormalities over time, consistent with a shift toward milder central nervous system involvement in congenital CMV infection. The presence of focal or multifocal white matter abnormalities in cUS decreased from 7.0% in P1 to 3.6% (*p* = 0.007) in P2, while the presence of single intracranial calcifications declined markedly from 7.9% to 1.6% (*p* < 0.001). The proportion of children with periventricular cysts declined from 11.0% to 6.1% (*p* = 0.002).

In contrast, the proportion of children presenting with caudothalamic or subependymal cysts (or germinolysis) increased significantly after 2017, from 3.1% to 13.5% (*p* < 0.001). Finally, the presence of multiple intracranial calcifications also increased in P2 (2.5% vs. 5.0%, *p* = 0.032).

## Cranial MRI

The proportion of children who underwent cranial MRI increased, from 48% before 2017 (269/566) to 65% after 2017 (711/1087). Cranial MRI findings at initial assessment were less severe in P2. The proportion of infants with entirely normal MRI findings increased from 36.1% in the P1 to 49.5% in P2 (*p* < 0.001), underscoring a shift toward less severe structural brain involvement.

The focal or multifocal white matter abnormalities were described in 39.8% of P1 and 21.5% of P2 (*p* < 0.001). Similarly, single intracranial calcifications decreased from 10.4% to 1.7% (*p* < 0.001). Mild to moderate ventriculomegaly (defined as > 97th centile) also declined, from 13.0% to 7.7% (*p* = 0.015). The proportion of newborns with periventricular cysts also decreased in P2 (18.2% vs. 9.4%, *p* < 0.001). Notably, cortical migration abnormalities, such as lissencephaly, were also less commonly observed in the later cohort, decreasing from 8.6% to 1.5% (*p* < 0.001).

## Treatment

Antiviral treatment was administered to 62.3% of children in P1, compared to 47.8% in P2 (*p* < 0.001). Among treated children, the choice of antiviral agent shifted over time. Use of intravenous ganciclovir decreased significantly (from 7.3% to 2.8%, *p* < 0.001), and its duration was also shortened (median 18.0 days to 12.0 days, *p* < 0.001). In contrast, the use of valganciclovir alone increased from 43.8% to 78.7%, while treatment with both ganciclovir and valganciclovir decreased from 48.9% to 18.6% (*p* < 0.001). Median treatment duration with oral valganciclovir slightly increased, from 162.0 days to 181.0 days (*p* < 0.001).

## Follow-up visits

At 6 months, motor impairment (paresis or spasticity) was significantly less frequent in the later cohort (14.1% vs. 6.1%, *p* < 0.001). Any form of hearing loss (mild/moderate/severe/profound) was observed in 29.3% of children in P1 and in 24.3% in P2, *p* = 0.072. The proportion of children with any long-term sequelae at 6 months decreased from 32.1% to 25.5% (*p* = 0.027).

At 12 months, improvements were more pronounced. Hearing loss was significantly less common in the later cohort (32.9% vs. 25.3%, *p* = 0.01), and motor impairment decreased substantially (16.5% vs. 6.2%, *p* < 0.001). Epilepsy under treatment was reported in 4.1% of children before 2017 compared to 1.8% in P2 (*p* = 0.043), while visual impairment (including chorioretinitis, optic atrophy, or cortical visual loss) declined from 3.9% to 1.5% (*p* = 0.027). Overall, the percentage of children with any long-term sequelae declined from 38.9% to 26.9% (*p* < 0.001).

The presence of long-term sequelae significantly decreased in children born in P2 when compared to P1 in both 24 months (37.4% to 29.3%, *p* = 0.026) and 36-month evaluation (40.0% to 31.0%, *p* = 0.05) (Table 1).

## Discussion

This retrospective, multicenter analysis of children with congenital cytomegalovirus (cCMV) infection across Europe revealed significant temporal changes in clinical presentation, management, and outcomes when comparing two periods: 2011–2016 and 2017–2024. These temporal changes occurred alongside evolving diagnostic, imaging, and treatment practices [7–9]. The European expert consensus statement published in 2017 provides an important context for interpreting these changes, and the most recent ECCI guidelines are also expected to further influence future management [10].

Interestingly, some findings—particularly caudothalamic or subependymal cysts—were reported more frequently after 2017. This may be explained by a higher proportion of children with mild disease. Taken together, these findings likely indicate a temporal trend toward a lower diagnostic threshold and fewer pathological findings on neuroimaging. The observed changes in clinical presentation may partly reflect changes in the pathways leading to cCMV diagnosis. In the later period, maternal seroconversion and newborn screening, including failed newborn hearing screening, accounted for a larger proportion of diagnoses, whereas diagnosis prompted by hearing loss became less frequent (Table 2). These findings indicate that the pathways through which infants with cCMV were identified changed over time and suggest that changes in antenatal and neonatal detection may have contributed to the identification of a broader spectrum of infants with cCMV, including those with milder manifestations. However, the present data do not allow us to determine whether these changes directly contributed to the observed differences in clinical presentation.

Changes in antiviral treatment with a higher proportion of children only receiving oral antivirals occurred during a period of evolving evidence and recommendations [10], which support the use of the oral route in most children. Extended antiviral treatment for 6 months was recommended after the publication of an RCT in 2015, which showed that extending oral valganciclovir therapy from 6 weeks to 6 months improved long-term hearing and neurodevelopmental outcomes in symptomatic infants [11]. Together, these changes are consistent with a shift towards more individualized, evidence-informed care, in line with updated recommendations and growing clinical experience.

Severe long-term sequelae have shown marked improvement across all follow-up intervals, with a consistently higher proportion of children in the post-2017 cohort experiencing no sequelae. Compared to the earlier cohort (2011–2016), children in the later cohort (2017–2024) exhibited significantly lower rates of motor impairment, epilepsy, and visual and hearing deficits. These findings raise the hypothesis that earlier and more precise diagnosis, together with evolving treatment strategies, may have contributed to improved long-term outcomes. However, the reasons underlying these differences cannot be determined from our registry data, and the observational design does not allow causal relationships to be established. This hypothesis warrants further investigation in studies specifically designed to evaluate the impact of changes in diagnostic and treatment strategies on long-term outcomes.

Although the newly published 2024 European Clinical Consensus Initiative (ECCI) guideline on the diagnosis and management of congenital CMV marks a significant step toward harmonizing care across Europe, its release during the year 2024 means it is unlikely to have substantially influenced the data analyzed in this study. Nevertheless, it is expected to further strengthen early detection efforts, unify treatment approaches, and support broader implementation of both targeted and universal screening strategies.

Study limitations include the retrospective observational design, possible reporting bias, missing data on antenatal treatment and follow-up in some cases, and heterogeneity in screening, diagnostic, and clinical practices across participating centers.

Differences in diagnostic pathways and incomplete information on how individual infants came to be tested may limit the interpretation of temporal changes in clinical presentation and outcomes. In addition, differences in center participation and case contribution between the two study periods may have contributed to heterogeneity in the observed temporal trends. The registry did not systematically capture changes in local screening policies or the implementation of recommendations at individual centers, limiting our ability to assess their contribution to the observed temporal changes. Similarly, indications for cranial imaging, parental choice, local availability of imaging, and specific imaging protocols were not systematically recorded, and no standardized radiological protocol or centralized image review was used across participating centers. These factors may have introduced selection bias and inter-center or temporal variability in the assessment and reporting of neuroimaging findings. Furthermore, the observational design precludes establishing causal relationships between changes in diagnostic or treatment practices and long-term outcomes.

As we move to an era where, in many European countries, prenatal diagnosis and secondary prevention with antivirals are offered to mothers with primary CMV infection, future prospective studies are needed to further evaluate temporal trends and assess the long-term benefits of early intervention strategies.

## Conclusion

This study highlights a temporal shift in the clinical spectrum, management, and outcomes of children with congenital CMV infection in Europe. A higher proportion of asymptomatic and mildly symptomatic children at birth in the latter period may reflect changes in maternal and neonatal screening practices. These findings provide insight into the evolving clinical landscape of cCMV diagnosis and management, although the underlying causes of these temporal differences cannot be determined from the present data.

## Data Availability

The datasets analysed during the current study are not publicly available due to ethical and privacy restrictions. Requests for access to the data may be considered by the cCMVnet upon reasonable request and in accordance with applicable ethical and data protection requirements.
